# Efficacy and Safety of Belatacept Treatment in Renal Allograft Recipients at High Cardiovascular Risk—A Single Center Experience

**DOI:** 10.3390/jcm8081164

**Published:** 2019-08-03

**Authors:** Hannes Neuwirt, Irmgard Leitner-Lechner, Julia Kerschbaum, Michael Ertl, Florian Pöggsteiner, Nicolas Pölt, Julius Mätzler, Hannelore Sprenger-Mähr, Michael Rudnicki, Peter Schratzberger, Iris E. Eder, Gert Mayer

**Affiliations:** 1 Department of Internal Medicine IV-Nephrology and Hypertension, Medical University of Innsbruck, Anichstrasse 35, Innsbruck A-6020, Austria; 2 Department of Pharmacology, Section for Molecular and Cellular Pharmacology, Medical University of Innsbruck, Peter-Mayr-Strasse 1a, Innsbruck 6020, Austria; 3 Landeskrankenhaus Feldkirch, Department of Internal Medicine III—Nephrology, Carinagasse 47, Feldkirch 6800, Austria; 4 Department of Urology, Medical University of Innsbruck, Anichstrasse 35, Innsbruck A-6020, Austria

**Keywords:** kidney transplantation, Belatacept, cardiovascular high risk, outcome

## Abstract

Belatacept is an attractive option for immunosuppression after renal transplantation. Renal allograft function is superior when compared to calcineurin inhibitor (CNI) based therapy in “de novo” treated patients and it has also been proposed that individuals at high cardiovascular (CV) risk may benefit most. In this retrospective cohort study, we assessed the efficacy and safety of treating patients at high cardiovascular risk with Belatacept (*n* = 34, for 1194 observation months) when compared to a matched control group of 150 individuals under CNI immunosuppression (for 7309 months of observation). The estimated glomerular filtration rate (eGFR) increased for patients taking Belatacept but decreased during CNI-based therapy (+2.60 vs. −0.89 mL/min/1.73 m^2^/year, *p* = 0.006). In a multivariate Cox regression model, Belatacept remained the only significant factor associated with the improvement of eGFR (HR 4.35, 95%CI 2.39–7.93). Belatacept treatment was not a significant risk factor for renal allograft rejection or graft loss. In terms of safety, the only significant risk factor for de novo cardiovascular events was a pre-existing cerebrovascular disease, but Belatacept was not associated with a significant risk reduction. Belatacept treatment was not associated with an increased risk of severe infections, cytomegalo virus (CMV) or BK-virus reactivation, malignancy or death in the multivariate Cox regression analysis. Belatacept is an efficient and safe option for patients after renal transplantation at high cardiovascular risk.

## 1. Introduction

Calcineurin inhibitors (CNIs) are currently the standard immunosuppressive therapy after renal transplantation. Their introduction into clinical practice has improved short-term outcomes dramatically. Unfortunately, the rate of late allograft loss has not significantly improved [1] and it is generally accepted that CNI nephrotoxicity contributes to this problem. Thus, multiple studies have investigated the impact of CNI-free immunosuppression on renal allograft function and patient and graft survival. The use of mammalian target of rapamycin inhibitors (mTORi) is impeded by drop-out rates of up to 40% due to side effects [2] and furthermore is associated with higher allograft rejection rates [3].

Belatacept inhibits T-cell activation by blocking a co-stimulatory signal by binding to CD80/CD86 on antigen presenting cells [4]. It is currently approved for the de novo immunosuppression of renal allograft recipients in combination with mycophenolic acid and steroids. Studies have demonstrated an improved allograft survival over several years when compared to cyclosporine A-based immunosuppression [5,6]. Furthermore, a protocol for switching patients from CNIs to Belatacept has been published [7,8]. This conversion improved kidney function relative to the baseline and was safe concerning risk of death or transplant loss. Finally, it has been proposed that Belatacept-based regimens might have beneficial effects, especially in patients at high cardiovascular (CV) risk (reviewed by [9]). One mechanism might be a reduction of pulse wave velocity in patients treated with Belatacept compared to CNI-treated patients [10,11]. However, “real world” data on renal outcomes and especially safety in the latter individuals are sparse.

Thus, we conducted a retrospective cohort study in renal allograft recipients at high CV risk and compared the efficacy and safety of Belatacept treatment in 34 patients to the outcomes of 150 patients treated with CNI (mainly tacrolimus) based immunosuppression.

## 2. Materials and Methods

### 2.1. Patient Population

Belatacept has been used in 42 renal transplant recipients at our center since 2010 and in this retrospective cohort study, all patients at high cardiovascular risk (definition see below) have been included (*n* = 34). Eighteen patients were treated de novo and 16 were converted at a median of 1.6 months (interquartile range (IQR), 0.6–4.2 months) after transplantation, mainly due to biopsy confirmed or clinically suspected renal CNI toxicity. No patient in this group was returned to CNI therapy thereafter. As we were interested in studying the efficacy and safety in patients on either CNI or Belatacept therapy, the day of conversion was taken as the baseline in these individuals and all clinical endpoints were adjudicated to the Belatacept group. Due to the early conversion, we excluded the time on CNI from any calculation. The study was conducted in accordance with the Declaration of Helsinki, and the protocol was approved by the Ethics Committee of the Medical University Innsbruck (Project-ID: 1137/2019).

The median observation duration on Belatacept was 35 months, no patient was lost for follow-up and the total period on therapy analysed was 1194 months. All renal allograft recipients (*n* = 309) on CNI-based immunosuppression that received their transplant between 1 January, 2010 and 31 December, 2012 formed the control cohort. Of these, 150 also fulfilled the criteria for high cardiovascular risk. No patients were lost for follow-up, the median follow-up was 48 months and the total analysed period of months on therapy was 7309. High cardiovascular risk was defined by the presence of any significant pre-transplant coronary artery disease (CAD) confirmed on angiography, a history of myocardial infarction, peripheral artery disease (PAD) (cardiovascular disease) or stroke (cerebrovascular disease) or the presence of diabetes mellitus in combination with arterial hypertension.

### 2.2. Endpoints

Efficacy endpoints were renal allograft function as assessed by a change of eGFR on therapy, number of rejection episodes (either confirmed by biopsy or clinically based on an improvement of allograft function after anti-rejection therapy) or graft loss. The estimated glomerular filtration rate (eGFR) was calculated using the abbreviated MDRD formula. ΔeGFR was calculated by dividing the difference between eGFR at last follow-up and the baseline by the number of follow-up years. The safety endpoints were de novo cardiovascular events (new myocardial infarction, newly diagnosed CAD of any stage, newly diagnosed peripheral artery disease), severe infections (defined as infection leading to the admission of the patient to hospital), cytomegalo virus (CMV) reactivation (diagnosed by PCR with or without a clinical CMV infection), BKV reactivation (as determined by PCR in serum and/or urine), de novo malignancy and death. All efficacy and safety endpoints were identified using patients’ records.

### 2.3. Statistics

The reported values represent either medians and interquartile ranges (IQR) or the number of patients and percentages of the respective cohort. Proportions were compared using the Chi^2^ or Fisher exact tests. Non-parametric tests were used to compare continuous variables. The factors potentially associated with the eGFR, the eGFR-slope (eGFR), and efficacy and safety parameters were assessed using a Cox regression analysis. In particular, those factors were: Belatacept treatment, recipient age, male gender of the recipient, recipient BMI, a CMV high risk mismatch (D+/R-), the presence of diabetes mellitus or arterial hypertension, the presence of cerebrovascular or cardiovascular disease, the time on renal replacement therapy (RRT) before renal transplantation (RTx), number of previous RTx, number of HLA mismatches, intraoperative urine production (initial diuresis), number of post-operative (PO)—meaning after renal transplantation—hemodialysis sessions (HDs), the absence or presence of steroids at discharge, the presence of serum-creatinine at discharge, the absence or presence of steroids at the last follow-up, the extended criteria donor (ECD) organ, the male sex of the donor, and donor age. A history of rejection was also included, with an exception for the endpoint rejection episodes. Variables with a *p*-value < 0.05 in univariate analysis were included in the multivariate analysis, where again a *p*-value < 0.05 was considered statistically significant. The analysis was performed using SPSS Version 24.

## 3. Results

### 3.1. Baseline

Baseline data are shown in Table 1. Belatacept patients spent a shorter period of time on renal replacement therapy (RRT) before renal transplantation, were more likely to suffer from CV disease or hypertension and less likely to have diabetes mellitus compared to CNI patients. The baseline data were not significantly different between de novo and converted Belatacept patients, except for the higher proportion of male recipients in de novo patients (17/18 vs. 8/16, *p* = 0.003).

### 3.2. Renal Transplantation

Concerning renal transplantation (RTx, Table 2), the donor type was significantly different between CNI- and Belatacept-treated patients. This was primarily driven by a higher proportion of living donors and deceased donors that died due to circulatory reasons in the Belatacept group. The Belatacept patients received more organs from female donors and donors were older (61 vs 49.5 years) and had a higher BMI compared to CNI patients. The proportion of patients with intraoperative urine production (initial diuresis) was lower (76% vs. 95%) and the number of hemodialysis sessions (HDs) was significantly higher in Belatacept patients. Hence, renal allograft function at discharge, as assessed by serum creatinine (1.52 vs. 2.20 mg/ml, *p* = 0.001) and eGFR (MDRD) (44.5 vs. 28 mL/min/1.73 m^2^, *p* = 0.001), was significantly worse in Belatacept patients.

### 3.3. Efficacy

The median follow-up (Table 3) was 1462 and 1054 days in CNI and Belatacept patients (*p* = 0.084), respectively. Belatacept was continued in all patients with a maintained graft function during follow-up (31/34). The number of patients on steroids at follow-up and the proportion of hypertensive patients were higher in the Belatacept group.

Concerning efficacy, renal allograft function as assessed by serum creatinine/eGFR improved in Belatacept-treated patients and slightly worsened in CNI patients, yielding a non-significant difference between the groups at follow-up (in contrast to the time of discharge after RTx). ΔeGFR of the patients on Belatacept was positive compared to CNI patients (+2.60 vs. −0.89 mL/min/1.73 m^2^/year, *p* = 0.006). The median ΔeGFR in the whole cohort (Belatacept plus CNI patients, *n* = 184) was + 0.35 mL/min/1.73 m^2^ (Table 4). The only factor significantly associated with a ΔeGFR above the median in the multivariate model (adjusted for significant factors in the univariate analysis including Belatacept treatment, recipient BMI, number of postoperative HDs, the presence of serum-creatinine at discharge and donors’ age) was Belatacept treatment (HR 4.35, 95%CI 2.387–7.926, *p* < 0.001, Table 4). Rejection episodes and graft loss were not significantly different between Belatacept and CNI patients (Table 5). Neither Belatacept nor any other parameter was a significant risk factor for rejection in the univariate Cox regression. Univariate correlated risk factors for graft loss were a higher recipient BMI (HR 1.126, 95%CI 1.038–1.222, *p* = 0.004), number of postoperative HDs (HR 1.178, 95%CI 1.041–1.333, *p* = 0.009) and higher serum-creatinine at discharge (HR 1.598, 95%CI 1.179–2.166, *p* =0.03). BMI (HR 1.116, 95%CI 1.003–1.242, *p* = 0.043) and number of postoperative HDs (HR 1.253, 95%CI 1.027–1.530, *p* = 0.027) remained significant after multivariate adjustments. Belatacept was not a risk factor for graft loss (HR 0.987, 95%CI 0.283–3.417, *p* = 0.980).

### 3.4. Safety

**Figure 1:**
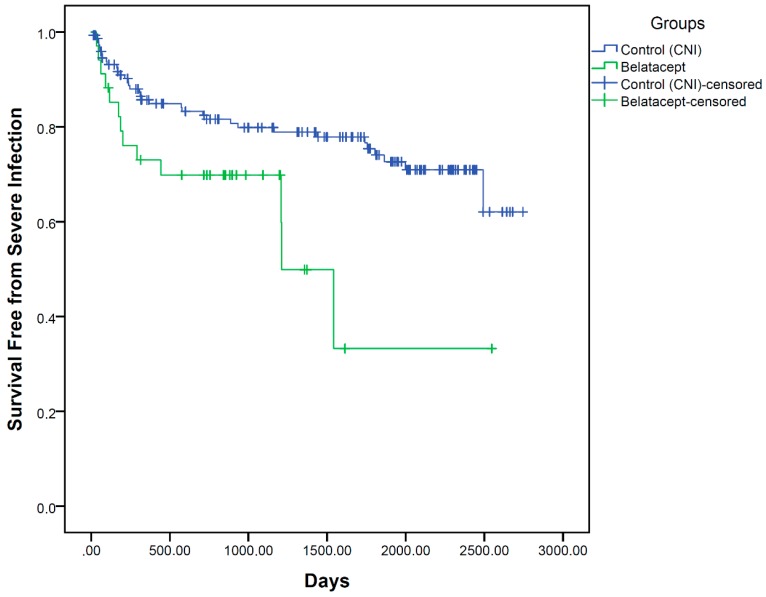
KM-Plot for severe infection (Log-Rank *p* = 0.013), defined as infection leading to the admission of the patient to hospital.

We found no difference between CNI- and Belatacept-treated patients concerning all safety endpoints, except for severe infection (Figure 1, Table 5).

Concerning severe infections, defined as infections leading to the admission of the patient to hospital, we found more infections in the Belatacept group (38.2 vs. 23.3%, Log-Rank *p* = 0.013). The type of severe infection also differed between the groups, with a higher proportion of diarrhoea, urinary tract infections and sepsis, but fewer instances of pneumonia, in Belatacept-treated patients. In the univariate Cox regression analysis, Belatacept treatment, number of postoperative HDs, the presence of creatinine at discharge, ECD and donor age were significant risk factors for severe infections (Table 6), while the male sex of the recipient was a protective factor. In the multivariate analysis, no risk factor remained significant (including Belatacept), whereas the male sex of the recipient remained a significant protective factor for severe infection in our cohort. It is noteworthy that Belatacept was not a significant risk factor in any Cox regression analysis for all other safety endpoints. Risk factors for CMV reactivation were number of postoperative HDs (HR 1.123, 95%CI 1.050–1.201, *p* = 0.001) and the presence of serum-creatinine at discharge (HR 1.335, 95%CI 1.087–1.638, *p* = 0.006) of which none remained significant in a multivariate model. A risk factor for BKV reactivation in patients’ plasma was treatment with steroids at follow-up (HR 3.358, 95%CI 1.246–9.051, *p* = 0.017) whereas the male sex of the donor was protective (HR 0.362, 95%CI 0.142–0.917, *p* = 0.032). The treatment with steroids at follow-up remained significant in the multivariate model (HR 2.850, 95%CI 1.042–7.796, *p* = 0.041). BKV reactivation in patients’ urine was significantly correlated with recipient (HR 1.041, 95%CI 1.013–1.069, *p* = 0.004) and donor age (HR 1.020, 95%CI 1.001–1.038, *p* = 0.036), of which recipient age remained multivariately significant (HR 1.033, 95%CI 1.003–1.064, *p* = 0.029).

The only univariate risk factor for the safety endpoint de novo cardiovascular events was pre-existing cerebrovascular disease (HR 6.144, 95%CI 1.026–36.798, *p* = 0.047). All other parameters, and especially Belatacept (HR 1.938, 95%CI 0.214–17.591, *p* = 0.557), were not significant. Concerning malignancy, we found no significant factor in the univariate Cox regression. Univariate risk factors for death were recipient age (HR 1.044, 95%CI 1.006–1.083, *p* = 0.023), HLA mismatch (HR 2.27, 95%CI 1.011–5.099, *p* = 0.047) and the presence of creatinine at discharge (HR 1.350, 95%CI 1.018–1.791, *p* = 0.037). Recipient age was the only significant risk factor for death in multivariate Cox regression (HR 1.036, 95%CI 1.001–1.074, *p* = 0.046). 

## 4. Discussion

We conducted a retrospective cohort study of renal allograft recipients at high cardiovascular risk either treated with a Belatacept- or CNI-based immunosuppressive regimen. eGFR improved with Belatacept treatment, but slightly decreased during CNI therapy and, in the multivariate analysis, Belatacept treatment was the only significant factor for the improvement of ΔeGFR. This is in line with the BENEFIT and BENEFIT-EXT studies, which also demonstrated an increase of GFR over a follow-up of seven years [12,13,14]. However, the CNI comparator cohort consisted of cyclosporine A-treated patients only, whereas our group was mainly treated with tacrolimus (68% of patients). Our recipients were of a similar age (56 years) to recipients in BENEFIT-EXT, but older compared to BENEFIT (43 years). Additionally, our CV high risk cohorts consisted of more diabetic patients (CNI group: 57%, Belatacept group: 32% vs. BENEFIT: 15%, BENEFIT-EXT: 16%) and had worse donor characteristics (living donors: CNI group 4%, Belatacept group 15% vs. BENEFIT 58%, BENEFIT-EXT not reported). Furthermore, the BENEFIT studies did not report the number of patients suffering from established cardiovascular disease, which was substantial in our Belatacept (94%) and CNI patients (75%). Nevertheless, and although Belatacept patients had inferior renal allograft function at the time of discharge after transplantation, serum creatinine levels and eGFR were similar at follow-up in this high CV risk cohort compared to CNI-treated patients. Bertrand et al. [15] and Le Meur et al. [16] reported similar results in 17 and 25 patients treated with Belatacept, because of vascular damage and CNI intolerance.

Belatacept was not associated with an increased risk of rejection in our patients. BENEFIT-EXT [17] reported a higher risk in Belatacept-treated patients, whereas BENEFIT [18] found no significant difference. However, our CNI cohort was mainly treated with tacrolimus, which is generally accepted to have a slightly higher immunosuppressive potential, rather than cyclosporine A as in the BENEFIT(-EXT) studies. Belatacept was not a risk factor for graft loss in our cohort (HR 0.987, 95%CI 0.283–3.417, *p* = 0.980), which is in line with the literature [17,18].

Concerning safety and in contrast to Florman et al. [13], we found that Belatacept treatment was associated with an increased incidence of severe infections in the univariate Cox regression. The most obvious differences were higher event rates of diarrhoea, urinary tract infection and sepsis. Sepsis as an endpoint has not been reported in any previous studies of Belatacept patients. In line with our data, gastroenteritis and urinary tract infections were numerically higher in the switch studies [7,8,19]. However, in the multivariate regression analysis, Belatacept treatment was not associated with an increase of this endpoint. One explanation for the higher incidence of gastroenteritis (diarrhoea) might be the more frequent use of mycophenlate in Belatacept patients (76.47 vs. 60.67%; 1.26–fold more), which is known to have such gastrointestinal side-effects. The reason for this is that physicians tend to prescribe the triple combination of mycophenolic acid, steroids and Belatacept, as only this regimen was approved for renal allograft recipients. However, it cannot be ruled out that some examples of mycophenolate-associated diarrhoea have been misdiagnosed as infectious diarrhoea, therefore increasing the proportion of diarrhoea in Belatacept patients compared to CNI patients, although the fold-change of diarrhoea (8.8 vs. 0.7%, 12.57-fold) substantially exceeds the use of mycophenlate in the Belatacept compared to the CNI group (1.26-fold, see above). Additionally, the proportion of mycophenolate-treated patients in the switch studies was almost identical between the groups (and higher compared to our data (approx. 94%)) [7,8,19].

In our cohort, Belatacept treatment was not a risk factor for CMV reactivation, malignancy or death. This is in line with the published data cited above. Additionally, Belatacept treatment was not a risk factor for BKV reactivations either in patients’ serum or urine. Unfortunately, data on these endpoints were not reported in the BENEFIT and BENEFIT-EXT studies. Nevertheless, our data is in line with the phase II studies, which showed only slightly increased cumulative incidence rates (0.85 vs. 0 [19]) and events (4 vs. 0% [8] and 2 vs. 0% [7]) in Belatacept patients. Unfortunately, no statistics were calculated in these studies.

Published data suggest a beneficial impact of Belatacept on arterial stiffness and metabolic parameters (e.g., arterial hypertension and lipid profile) or post-transplant diabetes mellitus. The authors concluded that this could improve kidney transplant recipients’ survival by reducing events related to those factors [9,17,18,20]. However, available data from the long-term outcomes of these studies do not show a significant difference in severely adverse events (including cardiac or vascular disorders) [12,14]. Concerning patients with high cardiovascular risk, the only study that has been published so far was a post-hoc analysis of patients with pre-existing diabetes of the BENEFIT and BENEFIT-EXT cohorts. Patient survival and renal function were numerically but not significantly higher in Belatacept patients at 12 months’ follow-up and, unfortunately, cardiovascular events were not reported [8]. Hence, to the best of our knowledge, this is the first report on cardiovascular events in Belatacept compared to CNI-treated patients. We found no difference between Belatacept- and CNI-treated patients concerning de novo cardiovascular events with a cumulative follow-up of 1194 months in Belatacept (*n* = 35) and 7309 months in CNI patients (*n* = 150).

Our study has limitations. Firstly, this is a retrospective study, which by nature does not provide the same data quality as a prospective design. Secondly, the size of the study population is relatively small, as we included only 34 Belatacept patients and 150 CNI patients as a comparator. Thirdly, the baseline characteristics of time on RRT, primary renal disease, diabetes, cardiovascular disease and arterial hypertension were different between our two populations (Table 1) and it is possible that statistical methods were not able to correct appropriately for this issue. Fourthly, the median time of follow-up was longer in CNI patients (Belatacept: 1054 vs. CNI: 1462 days) but not statistically significant (*p* = 0.084). From our point of view, the duration of follow-up is still significant, although one might argue that a longer follow-up would have been beneficial, especially for the end point “cardiovascular event”. However, the number of studies that have published data of renal allograft recipients on Belatacept-based immunosuppression is generally limited. In total, until the end of 2014, the data of 521 Belatacept patients, which were compared to CNI-treated controls, were published [21]. Recently, one study of 17 Belatacept patients matched to 18 control patients, and two studies of 25 and 6 cases that were converted from CNI to Belatacept without a control population, were published [15,16,22]. The randomized controlled trials BENEFIT [18], BENEFIT-EXT [17] and the switch study [8] originally reported one-year results of 181, 129 and 81 belatacept patients compared to a CNI-treated cohort of similar size. Hence, we believe that our cohort size and follow-up period is considerable and contributes information in a real world setting.

In conclusion, we believe that Belatacept is an efficient, beneficial and safe option for renal allograft recipients at high cardiovascular risk. In our cohort, Belatacept treatment was associated with a superior graft function compared to a CNI-treated cohort and was not a risk factor for renal allograft rejection, -loss, severe infection, CMV- or BKV-reactivation, malignancy or death. 

## Figures and Tables

**Table 1:** Baseline data of control and Belatacept patients.

	Control (*n* = 150)		Belatacept (*n* = 34)		
	Median/*n*	IQR/(%)	Median/*n*	IQR/(%)	*p*
BMI [kg/m^2^]	25.1	22.6–28.4	26.1	22.6–30.6	0.304
Male Sex *n* (%)	37	(24.67)	9	(26.47)	0.826
Age at time of RTx [years]	59.6	49.4–66.8	57.2	38.7–65.4	0.325
Time on RRT [months]	53.8	29.6–80.5	35.6	22.2–52.1	0.006 *
Primary Renal Disease *n* (%)					
Diabetic Nephropathy	51	(34.00)	5	(14.71)	0.031 *
Vascular Nephropathy	25	(16.67)	5	(14.71)	0.768
IgA Nephropathy	7	(4.67)	6	(17.65)	0.017 *
other Glomerulonephritis	18	(12.00)	2	(5.88)	0.377
ADPKD	12	(8.00)	5	(14.71)	0.321
other hereditary disease	2	(1.33)	0	(0.00)	1.000
ANCA Vasculitis	0	(0.00)	0	(0.00)	n.c.
Lupus nephropathy	2	(1.33)	0	(0.00)	1.000
chronic kidney disease NOS	33	(22.00)	11	(32.35)	0.209
Number of previous RTx *n* (%)					0.671
0	114	(76.00)	29	(85.29)	
1	24	(16.00)	4	(11.76)	
2	10	(6.67)	1	(2.94)	
3	1	(0.67)	0	(0.00)	
4	1	(0.67)	0	(0.00)	
Diabetes *n* (%)	85	(56.67)	11	(32.35)	0.009 *
Cerebrovascular disease *n* (%)	12	(8.00)	4	(11.76)	0.503
Cardiovascular disease *n* (%)	113	(75.33)	32	(94.12)	0.016 *
Arterial Hypertension *n* (%)	126	(84.00)	34	(100)	0.014 *

The median and interquartile range (IQR) are depicted except for nominal variables, where the number of patients (*n*) and percentages are shown. *p*-values < 0.05 are marked with an asterisk *. BMI: body mass index, RTx: renal transplantation, RRT: renal replacement therapy, ADPKD: autosomal dominant polycystic kidney disease, ANCA: antineutrophil cytoplasmic antibody, NOS: not otherwise specified, n.c.: not calculated.

**Table 2:** Data at the time of Renal Transplantation (RTx).

	Control (*n* = 150)		Belatacept (*n* = 34)		
	Median/*n*	IQR/(%)	Median/*n*	IQR/(%)	*p*
Donor Type *n* (%)					
Living donor	6	(4.00)	5	(14.71)	0.032 *
DD (CVA/SAB/SDH)	86	(57.33)	22	(64.71)	0.431
DD (trauma)	41	(27.33)	0	(0.00)	0.001 *
DD (circulatory)	6	(4.00)	5	(14.71)	0.032 *
DD (suicide)	7	(4.67)	2	(5.88)	0.673
DD (other)	4	(2.67)	0	(0.00)	1.000
ECD *n* (%)	65	(43.33)	18	(52.94)	0.309
Male Donor Sex *n* (%)	80	(53.33)	9	(26.47)	0.005 *
Donor Age [years]	49.5	37–66	61	51–68	0.003 *
Donor BMI [kg/m^2^]	24.9	22.5–27.7	26.3	24.5–28.6	0.034 *
CMV mismatch *n* (%)					0.947
Donor-/Recipient-	20	(13.33)	5	(14.71)	
Donor-/Recipient+ or Donor+/Recipient+	100	(66.66)	22	(64.71)	
Donor+/Recipient-	30	(20.00)	7	(20.59)	
Number of HLA Mismatches	3	3–5	3	2–5	0.843
Initial Diuresis *n* (%)	143	(95.33)	26	(76.47)	<0.001 *
Number of PO HDs after RTx	0	0–2	1	0–5	0.015 *
On Steroids at discharge *n* (%)	141	(94.00)	34	(100.00)	0.214
S-Creatinine at discharge (mg/dL)	1.52	1.21–2.11	2.20	1.40–2.98	0.001 *
eGFR at discharge (MDRD) (mL/min/1.73 m^2^)	44.5	30–9	28	22–51	0.001 *

Median and IQR are depicted except for nominal variables, where the number of patients (*n*) and percentages are shown. *p*-values < 0.05 are marked with an asterisk *. DD: deceased donor, CVA: cerebrovascular event, SAB: subarachnoidal bleeding, SDH: subdural hematoma, ECD: extended criteria donor, CMV mismatch: “−” means sero-negative, “+” means sero-positive, PO HDs: postoperative hemodialysis sessions, RTx: renal transplantation, S-Creatinine: serum creatinine.

**Table 3:** Data at last follow-up.

	Control (*n* = 150)		Belatacept (*n* = 34)		
	Median/*n*	IQR/(%)	Median/*n*	IQR/(%)	*p*
Follow-up [days]	1462	718–2115	1054	772–1363	0.084
IS-CNI/mTor/Bela *n* (%)					<0.001 *
none	14	9.33	3	8.82	
Tac	105	70.00	0	0.00	
CsA	25	16.67	0	0.00	
Bela	0	0.00	31	91.17	
mTORi	6	4.00	0	0	
IS-Antimetabolites *n* (%)					0.221
none	32	21.33	5	14.71	
Mycophenolate Mofetil (MMF)	75	50.00	19	55.88	
Mycophenolic acid (MPA)	16	10.67	7	20.59	
Azathioprine	25	16.67	2	5.88	
other	2	1.33	1	2.94	
IS-On steroids *n* (%)	71	47.33	28	82.35	<0.001 *
Serum Creatinine (mg/dL)	1.62	1.25–2.36	1.96	1.342.31	0.404
eGFR (MDRD) (mL/min/1.73m^2^)	41	27–60	36.5	25–52	0.329
ΔeGFR (MDRD) (mL/min/1.73m^2^/year)	−0.89	−6.05–3.02	2.6	−1.85–7.76	0.006*
Cerebrovascular disease *n* (%)	13	8.67	5	14.71	0.335
Cardiovascular disease *n* (%)	118	78.67	33	97.06	0.004 *
Arterial Hypertension *n* (%)	120	80.00	30	88.24	1.000

Median and IQR are depicted except for nominal variables, where the number of patients (*n*) and percentages are shown. *p*-values < 0.05 are marked with an asterisk *. Data on cerebro- and cardiovascular diseases show cumulative numbers of events at follow-up. De novo events are depicted in Table 5. IS-CNI/mTOR/Bela: immunosuppression concerning tacrolimus (Tac), cyclosporine A (CsA), Belatacept (Bela), mTOR inhibitors (mTORi). IS-Antimetabolites: immunosuppression concerning mycophenolate mofetil (MMF), mycophenolic acid (MPA), azathioprine (Aza).

**Table 4:** Cox regression for eGFR better than the median (+0.35 mL/min/1.73 m^2^/year) at last follow-up. We calculated univariate hazard ratios (HR) for risk factors. All significant univariate risk factors were used in the multivariate model. BMI: body mass index, RTx: renal transplantation, PO HD: postoperative hemodialysis session, ECD: extended criteria donor.

Factor	Univariate Analysis			Multivariate Analysis ^1^		
	HR	95%-CI	*p*	HR	95%-CI	*p*
Belatacept	5.629	3.239–9.783	<0.001 *	4.350	2.387–7.926	<0.001 *
Recipient Age	1.012	0.994–1.030	0.184			
Male recipient Sex	1.435	0.863–2.388	0.164			
BMI	1.063	1.018–1.111	0.006*	1.024	0.979–1.072	0.300
Diabetes	0.799	0.524–1.218	0.297			
Cerebrovascular disease	1.365	0.704–2.646	0.357			
Cardiovascular disease	1.050	0.624–1.767	0.853			
Arterial hypertension	0.846	0.475–1.508	0.572			
Number of previous RTx	0.741	0.499–1.098	0.135			
HLA mismatch	0.649	0.416–1.011	0.056			
Initial Diuresis	0.502	0.200–1.260	0.142			
Number of PO HDs	1.117	1.051–1.187	<0.001 *	1.037	0.953–1.129	0.395
Steroid at discharge	2.424	0.760–7.731	0.134			
Creatinine at discharge	1.437	1.238–1.667	<0.001 *	1.218	0.885–1.675	0.226
ECD	1.357	0.891–2.066	0.154			
Male Donor Sex	1.131	0.742–1.725	0.566			
Donor Age	1.018	1.005–1.032	0.007 *	1.004	0.990–1.019	0.546

^1^ adjusted for Belatacept treatment, BMI, number of postoperative (PO) hemodialysis sessions (HDs), eGFR at discharge and donor age. *p*-values < 0.05 are marked with an asterisk *.

**Table 5:** Efficacy and safety endpoints at last follow-up. *p*-values < 0.05 are marked with an asterisk *. CMV: cytomegalo virus, BKV: polyoma virus.

	CNI (*n* = 150)		Belatacept (*n* = 34)			
EFFICACY	*n*	%	*n*	%	*p*	Log-Rank
Rejection	13	8.7	4	11.8	0.524	0.295
Graft loss	17	11.3	3	8.8	1.000	0.980
**SAFETY**						
De novo CV events	5	3.33	1	2.94	1.000	0.550
Severe Infection	35	23.3	13	38.2	0.074	0.013*
Type of severe Infection					0.003 *	
none	115	76.7	21	61.8		
Diarrhea	1	0.7	3	8.8		
Urinary tract infection	15	10.0	6	17.6		
Pneumonia	15	10.0	1	2.9		
Sepsis	4	2.7	3	8.8		
Any CMV reactivation	60	40.0	16	47.1	0.450	0.932
BKV reactivation in serum	16	10.7	7	20.6	0.148	0.136
BKV reactivation in urine	37	24.7	10	29.4	0.567	0.718
Malignancy	2	1.3	0	0.0	1.000	0.650
Death	22	14.7	4	11.8	0.790	0.861
Cause of Death					0.921	
unknown	5	3.3	1	2.9		
Sepsis	7	4.7	2	5.9		
Cardiac	7	4.7	1	2.9		
Malignancy	2	1.3	0	0.0		
Stroke	1	0.7	0	0.0		

**Table 6:** Cox regression for severe infection. All the significant risk factors from the univariate Cox-Regression are shown and were included in the multivariate analysis. *p*-values < 0.05 are marked with an asterisk *.

Factor	Univariate Analysis			Multivariate Analysis ^1^		
	HR	95%-CI	*p*	HR	95%-CI	*p*
Belatacept	2.236	1.163–4.301	0.016 *	1.363	0.659–2.819	0.403
Male recipient Sex	0.548	0.305–0.983	0.044 *	0.459	0.242–0.870	0.017 *
Number of PO HDs	1.156	1.062–1.259	0.001 *	1.057	0.939–1.189	0.358
Creatinine at discharge	1.602	1.255–2.044	<0.001 *	1.131	0.628–2.037	0.682
ECD	2.867	1.578–5.209	0.001 *	1.373	0.488–3.858	0.548
Donor Age	1.032	1.012–1.052	0.002 *	1.019	0.985–1.054	0.287

^1^ adjusted for belatacept treatment, male recipient sex, number of post-operative (PO) hemodialysis sessions (HDs), creatinine ad discharge, extended criteria donor (ECD), donor age.

## Abbreviations

BKV	polyoma virus
BMI	body mass index
CAD	coronary artery disease
CD	cluster of differentiation
CMV	cytomegalo virus
CNI	calcineurin inhibitor
CV	cardiovascular
CVA	cerebrovascular event
DD	deceased donor
ECD	extended criteria donor
eGFR	estimated glomerular filtration rate
HD	hemodialysis
HLA	human leukocyte antigen
HR	hazard ratio
IQR	interquartile range
mTORi	mammalian target of rapamycin inhibitor
PAD	peripheral artery disease
PCR	polymerase chain reaction
PO	post-operative after renal transplantation
RRT	renal replacement therapy
RTx	renal transplantation
SAB	subarachnoideal bleeding
SDH	subdural hematoma
95%CI	95% confidence interval
